# Keeping Safe: Intra-individual Consistency in Obstacle Avoidance Behaviour Across Grasping and Locomotion Tasks

**DOI:** 10.1177/2041669517690412

**Published:** 2017-02-01

**Authors:** Karina Kangur, Jutta Billino, Constanze Hesse

**Affiliations:** School of Psychology, University of Aberdeen, Scotland, UK; Experimental Psychology, Justus-Liebig-Universität Gießen, Germany; School of Psychology, University of Aberdeen, Scotland, UK

**Keywords:** vision, action, mobile eye tracking, locomotion, inter-individual differences

## Abstract

Successful obstacle avoidance requires a close coordination of the visual and the motor systems. Visual information is essential for adjusting movements in order to avoid unwanted collisions. Yet, established obstacle avoidance paradigms have typically either focused on gaze strategies or on motor adjustments. Here we were interested in whether humans show similar visuomotor sensitivity to obstacles when gaze and motor behaviour are measured across different obstacle avoidance tasks. To this end, we measured participants’ hand movement paths when grasping targets in the presence of obstacles as well as their gaze behaviour when walking through a cluttered hallway. We found that participants who showed more pronounced motor adjustments during grasping also spent more time looking at obstacles during locomotion. Furthermore, movement durations correlated positively in both tasks. Results suggest considerable intra-individual consistency in the strength of the avoidance response across different visuomotor measures potentially indicating an individual’s tendency to perform safe actions.

## Introduction

Humans show a remarkable ability to avoid collision with obstacles when moving through a complex environment. As successful obstacle avoidance crucially depends on vision to accurately plan and control limb movements, it comes as no surprise that it is frequently used as an experimental paradigm to understand visuomotor integration (for a review see Higuchi, 2013; Rosenbaum, Meulenbroek, Vaughan, & Jansen, 1999). Obstacle avoidance is thereby predominately studied in two kinds of tasks: (a) reaching and grasping and (b) locomotion and stepping. While the two tasks differ in nature, they both reveal similar adjustment strategies. That is, if obstacles are present, participants slow down their actions and select movement paths that are safe and efficient. This is usually achieved by increasing the distance between the moving limb and the obstacles while keeping deviations away from the optimal path to a minimum (Chapman & Goodale, 2008; Patla, Tomescu, Greig, & Novak, 2007; Rosenbaum et al., 1999; Ross, Schenk, & Hesse, 2014). However, the accompanying gaze behaviour seems rather different for hand movement and locomotion tasks. While participants barely ever look at obstacles when performing hand movements in simple worktop-based setups (Johansson, Westling, Backstrom, & Flanagan, 2001; Ross, Schenk, & Hesse, 2015), they seem to purposefully gaze at obstacles that obstruct or clutter their movement path during locomotion for at least 20% of the time (Hayhoe, Gillam, Chajka, & Vecellio, 2009; Patla et al., 2007; Patla & Vickers, 1997). The lack of direct obstacle fixations in hand movement tasks possibly relates to the fact that obstacles are usually quite large and are placed in close vicinity to the target in order to maximise their effects on movement execution. Hence, they can easily be spotted in near periphery without shifting gaze.

Despite this apparent inconsistency between the two tasks, there is, however, evidence that gaze behaviour and motor adaptations are, in fact, closely linked processes in obstacle avoidance. For example, it was observed that individuals who show more cautious behaviour when avoiding obstacles during locomotion, such as senior individuals, spend more time looking at the obstacles suggesting that changes in gait are accompanied by changes in fixation behaviour (Chapman & Hollands, 2006; Santos, Araújo, & Moniz-Pereira, 2013). Conversely, there is evidence from a recent study employing a standard reaching paradigm, that the selected fixation locations affect motor adaptions, further implying a reciprocal relationship between gaze and motor behaviour during obstacle avoidance (Ross et al., 2015). Specifically, it was found that participants’ avoidance response tended to increase when they were instructed to directly gaze at an obstacle during reaching (i.e., larger distance between hand and obstacle). Yet, the exact mechanisms accountable for the enhanced avoidance response in the case of direct fixations are still unclear as alternations in gaze can generate both a perceptual as well as an attentional bias (for discussion see Ross et al., 2015). Indeed, Menger, Dijkerman, and Van der Stigchel (2015) have shown that increased attention toward an obstacle can be sufficient to enhance the avoidance response. In their study, participants were found to keep a larger distance from a flashing obstacle that captured attention more strongly than a nonflashing one. However, it is also important to point out that in natural tasks gaze behaviour and spatial attention are usually tightly coupled (Findlay & Gilchrist, 2003). Therefore, the attempt to experimentally dissociate these processes to determine their respective effects on obstacle avoidance behaviour may be a somewhat futile enterprise. Thus, in the current study, we assumed that during locomotion, gaze behaviour provides a good indicator for how much attention participants typically pay to obstacles placed in their environment.

Finally, it is also important to mention that the magnitude of the avoidance response is not only determined by the physical and spatial features of obstacles but also considerably depends on the associated consequences of potential collisions. This has been shown in both a hand-movement study (De Haan, Van der Stigchel, Nijnens, & Dijkerman, 2014) demonstrating that participants keep a larger distance away from an obstacle if the consequences of knocking it over are judged as being more severe (i.e., full water glass vs. empty water glass), as well as a locomotion study in which participants kept larger toe clearance when stepping over an obstacle that was perceived as being more fragile (Patla, Rietdyk, Martin, & Prentice, 1996). These observations, in combination with the above-reported finding that older adults who have a higher risk of falling with more dramatic consequences show larger avoidance responses (and increased obstacle fixations), let us speculate that the strength of the avoidance behaviour in general might be mediated by an individual’s inherent tendency to avoid potential risks.

We aimed to test this assumption by investigating whether individuals show similar responsiveness to obstacles when two quite different obstacle avoidance tasks are used. In other words, do people who pay more attention to obstacles in a relatively natural locomotion task, as indicated by increased obstacle fixations, also show larger motor adjustments in response to obstacles in a standard grasping task? This approach also allowed us to clarify if the proposed link between gaze behaviour and movement adjustments persists across tasks on an intra-individual basis.

## Results

**Figure 1.**
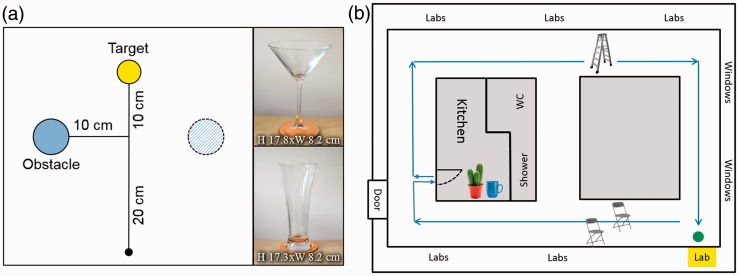
(a) Illustration of the grasping task. Participants grasped a wooden target cylinder while one obstacle (martini glass vs. lager glass) was placed either to the left or right of the midline (for more information, see Method section). (b) Illustration of the locomotion task. Two chairs, a stepladder, and a cactus served as obstacles when walking through the hallway (1.75 m wide, 65 m long) and getting a mug (that served as target) from a kitchen area.

**Figure 2.**
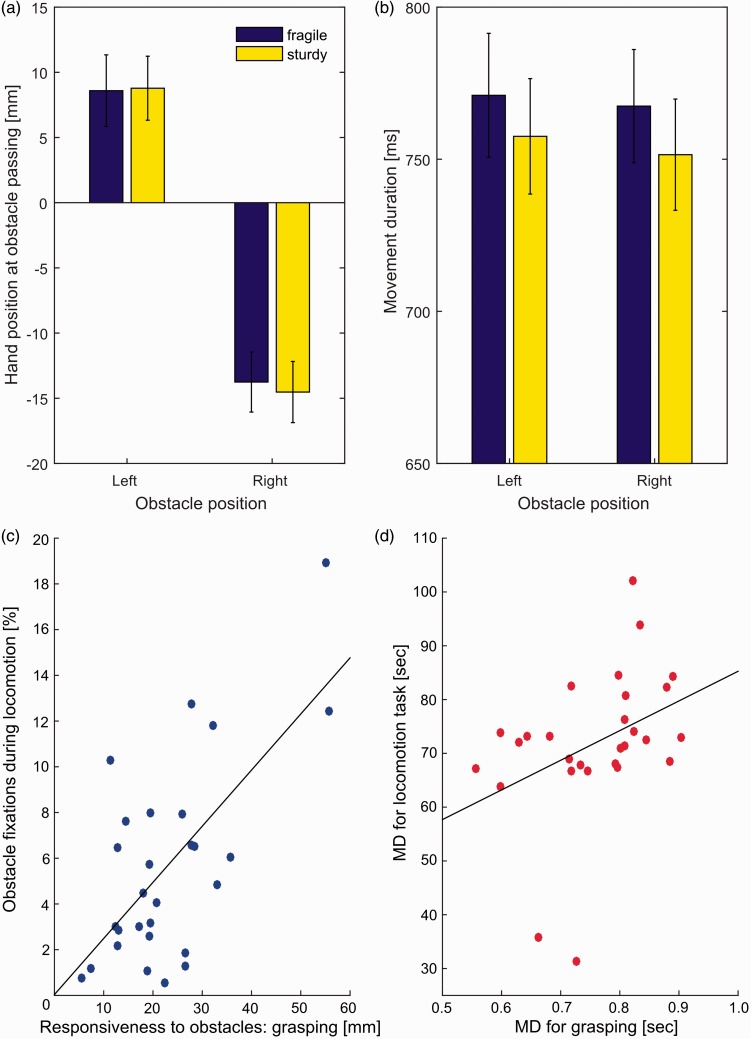
(a) Horizontal deviation of the hand away from the midline as a function of obstacle position and fragility when the obstacle was passed during grasping. (b) Movement duration for grasping as a function of obstacle position and fragility. Error bars depict ± 1 *SEM* (between-subjects). (c) Correlation between obstacle fixations in the locomotion task and the responsiveness to obstacles during grasping. (d) Correlation between the movement durations in the locomotion and the grasping task. Note that two participants completed the locomotion task in less than 35 seconds.

We used the paradigm illustrated in Figure 1(a) to study grasping in the presence of an obstacle. We determined movement duration (MD) and responsiveness to obstacles (i.e., the size of adjustments in hand position) as a function of obstacle position (left vs. right) and obstacle fragility (fragile vs. sturdy). Specifically, we predicted an enhanced avoidance response for the fragile glass as knocking it over is more likely to result in breakage and thus has stronger negative consequences associated with it (De Haan et al., 2014; Patla et al., 1996). A 2 (Obstacle position: left vs. right) × 2 (Obstacle fragility: fragile vs. sturdy) repeated-measures analysis of variance on the hand position data revealed an expected effect of obstacle side, *F*(1, 27) = 101.9, *p* < .001. On average, participants altered their hand position by about 23 mm depending on the obstacle’s position (Figure 2(a)). There was no effect of obstacle fragility (*p* = .56) indicating that participants selected similar movement paths for fragile and sturdy glasses. No interaction effect (*p* = .22) was present. However, the same analysis for MD showed that participants tended to move more slowly when the more fragile martini glass was placed on the table, *F*(1, 27) = 5.9, *p* = .02. This finding suggests that participants are slightly more cautious in this condition. On average, the movements took about 15 ms longer when the fragile glass served as an obstacle as compared with the more sturdy lager glass (see Figure 2(b)). In contrast, obstacle position had no effect on MD (*p* = .53), and there was no interaction effect (*p* = .74).

Our paradigm for measuring avoidance behaviour in a locomotion task is shown in Figure 1(b). To test if individuals’ responsiveness to obstacles and the accompanying visuomotor adjustments are comparable across hand movement and locomotion tasks, we determined for each participant the extent to which each of them adjusted their movement paths to the obstacle’s position in the grasping task and correlated this measure with the percentage of time they spent looking at obstacles in the locomotion task. Remarkably, we found a strong correlation between the two measures, *r*(28) = .664, *p* < .001 (see Figure 2(c)). Congruently, MDs in both obstacle avoidance tasks also correlated moderately, *r*(28) = .381, *p* = .046, indicating that participants who moved more slowly in the grasping task also took longer to navigate through the natural setting (Figure 2(d)). Note that there were, in fact, large inter-individual differences in how much time participants spent looking at obstacles during the locomotion task (from less than 1% of the movement time up to almost 20%) as well as in how long it took them to walk around the hallway (from just over 30 seconds up to about 1 minute 42 seconds).

Finally, we were interested in whether there is a relationship between participants’ MD and their responsiveness to obstacles within the grasping and locomotion tasks. Such a relationship could indicate individual differences in balancing speed and accuracy in the task. We expected faster movements to be associated with more pronounced obstacle avoidance behaviour (indicated by a larger proportion of obstacle fixations and movement path adjustments, respectively) due to increased safety requirements. We found that in the locomotion task, MD correlated negatively with the percentage of obstacle fixations, *r*(28) = −.404, *p* = .03, indicating that participants who moved faster tended to spend proportionally more time looking at obstacles. However, contrary to our expectations, we observed no relationship between MDs and responsiveness to obstacles in the grasping task, *r*(28) = −.084, *p* = .67. This is, in so far, surprising as a larger deviation away from a straight-line movement path (i.e., increased responsiveness to obstacles) should actually increase the movement distance, thereby prolonging MDs in turn. However, we speculate that due to the relative short movement path in the grasping task, variations in MD might have been too small to detect a reliable effect.

## Discussion

This study aimed to explore whether variations in visuomotor behaviour in response to obstacles during locomotion and grasping tasks are associated on an intra-individual basis. Our results suggest that while individuals strongly differ from one another in the magnitude of their behavioural response when confronted with obstacles, they show high intra-individual consistency in their visuomotor adjustments across different tasks and measures. We found a strong positive correlation between responsiveness to obstacles in grasping and obstacle fixations during locomotion. This implies that there are intra-individual differences in cautiousness with which participants plan and execute their movements which, in turn, may determine how much attentional weight individuals give to obstacles in their environment. In line with this argument, a recent study has shown that allocating more attention to obstacles (i.e., by flashing them) increases the strength of the avoidance responses (Menger et al., 2015).

While this is the first study that combines two quite distinct obstacle avoidance paradigms (i.e., grasping and locomotion tasks) in order to establish if participants show consistent levels of responsiveness to obstacles, both paradigms have been frequently employed previously in isolation (e.g., Chapman & Goodale, 2008; Menger, Van der Stigchel, & Dijkerman, 2012; Patla, 1997; Patla et al., 2007; Rosenbaum et al., 1999; Ross et al., 2014). Importantly, most of our results are well in line with previous observations such as participants adjusting their hand movements in response to obstacle position during grasping, and spending a considerable amount of time looking at obstacles prior to passing them during locomotion. Despite the similarities to earlier studies, there are also a few inconsistencies that need to be addressed.

First, we had predicted that participants will show greater deviations away from an obstacle during grasping if there are increased negative consequences associated with a potential collision. Evidence for this suggestion comes from a study by De Haan et al. (2014) who found that participants kept a larger distance from a full water glass as compared with an empty one in order to minimise the risk of spillage when performing hand movements. In our study, we used two different kinds of glasses that can be considered as being more or less fragile (martini vs. lager glass). Collision with a fragile glass can be deemed as being more dangerous as it is more likely to result in breakage. In contrast to this prediction, we found similar movement paths for both types of glasses. However, before concluding that perceived fragility of an obstacle had no effect on avoidance behaviour, we have to take into account that while we kept the maximum diameter of the two glasses constant, the Y-shape of the martini glass provided considerably more room for passing it when reaching for the target than the wider body of the lager glass (see Figure 1(a)). Hence, it seems almost surprising that participants still kept a similar distance from the martini glass as from the lager glass as this essentially means that there was a larger distance between the hand and the stem of the glass. Furthermore, in line with the idea that the martini glass is considered as the more *dangerous* (i.e., breakable) obstacle, we found that participants performed slower movements when it was placed in the workspace. This supports the findings of earlier studies showing that MDs get prolonged as obstacles become more obstructive and accuracy demands increase (Tresilian, 1998).

Second, with regard to locomotion task, one might wonder why we observed seemingly fewer and/or shorter fixations on obstacles as compared with previous studies (e.g., Hayhoe et al., 2009; Patla et al., 2007). Again, this can almost certainly be attributed to the differences in the task settings used in our experiment as compared with previous studies. For instance, in the locomotion study investigating participants’ gaze behaviour when navigating around obstacles, Patla et al. (2007) placed 12 obstacles (i.e., traffic pylons) in an area measuring about 4.5 × 3.2 m (resulting in walking path lengths of about 5–6 m). Other studies made the obstacles more obstructive, thereby requiring participants to actively avoid them by stepping over them (e.g., Hayhoe et al., 2009; Patla & Vickers, 1997). In contrast, in our experiment, participants walked a comparatively long path (about 65 m) in which we only placed a few obstacles. Hence, they spent overall a smaller percentage of their time looking at the obstacles. Using this less obvious obstacle avoidance setup in our experiment had, in fact, an advantage as our participants were actually unaware that we were interested in their obstacle avoidance performance (rather than in their navigation performance), thereby allowing us to measure more natural avoidance behaviour in this situation. Furthermore, Rothkopf, Ballard, and Hayhoe (2007) used a similar walking task within a virtual environment (40 m length, 1.8 m width) and showed that fixation patterns also strongly depend on the task instructions. That is, their participants spent a considerably smaller proportion of time fixating at obstacles placed in the walkway if they were instructed to pick up litter as compared with conditions in which they received explicit instruction on avoiding obstacles. Hence, the small percentage of obstacle fixations observed in our study might also be partly due to the fact that we did not mention obstacles at all in our task instruction.

In summary, we suggest that our grasping and locomotion paradigms allowed us to measure typical visuomotor adjustments when movements are challenged by obstacles—and that the extent of these adjustments may reflect the general tendency of an individual to perform safe actions. Specifically, our study provides first tentative evidence that participants who show larger avoidance responses in a simple grasping task also tend to allocate more attention to obstacles (as measured by gaze behaviour) in a more natural locomotion task. Based on these findings, one could speculate that inter-individual differences in obstacle avoidance behaviour may be the reason why some people bump into objects or knock their drinks over more frequently than others.

## Method

### Participants

Thirty participants with normal or corrected-to-normal vision gave informed consent prior to participating in the experiment that was approved by the Psychology Ethics Committee at Aberdeen University. Two participants were excluded from the analysis due to missing data in the video files from the locomotion task resulting in a total number of 28 participants (9 men, 18–36 years).

### Setup, Stimuli, and Procedure

In the grasping task, participants sat in a high-adjustable chair in front of a table on which the target object (a yellow cylinder with a height and width of 55 mm) and one of the obstacles (martini glass [fragile obstacle] vs. lager glass [fragile obstacle]; see Figure 1(a)) were placed. Participants’ hand movements were measured at 240 Hz with a TrakStar motion tracker (Northern Digital). Two markers were attached to the tip of the index finger and the tip of the thumb of the participants’ dominant hand. Participants wore liquid-crystal shutter glasses (Translucent Technologies, Milgram, 1987) to occlude vision during the time the experimenter arranged the objects on the table. In each trial, an obstacle was placed either 10 cm to the left or right of the movement path (i.e., straight-line distance of 30 cm between the start position and the target object). Once the obstacles were arranged in the workspace, the experimenter started each trial manually with a key press. At the start of the trial, the shutter glasses opened. Following a preview period of 1 seconds, an auditory go-signal was presented in response to which participants had to reach out, grasp the target object, and place it anywhere on the table. Participants had full visibility during movement execution (for 2 seconds after the go-signal) while shutter glasses were closed between trials to allow the experimenter to prepare the next trial. Each combination of obstacle position (left vs. right) and obstacle type (martini vs. lager glass) was tested 20 times in random order resulting in a total of 80 grasping trials.

In the locomotion task, the experimenter asked the participant to walk down a hallway into a kitchen area, get a coffee mug placed opposite to the sink and return it to the lab. Several obstacles were placed along the way (two chairs and a step ladder) and in the kitchen (a cactus) but were not noted in the instruction (see Figure 1(b)). Instead, the task was disguised as a brief pilot test for memory and navigation skills. Participants’ eye movements were tracked with SMI glasses (SensoMotoric Instruments) that recorded gaze at a sampling rate of 60 Hz with a spatial resolution of 0.1° and an accuracy of 0.5°. Furthermore, the surrounding was recorded via a scene camera at a rate of 24 Hz and with a resolution of 1280 × 960 pixels. Participants were informed about the scene camera but remained unaware of their eye movements being recorded. They were fully debriefed afterwards and were offered the possibility to withdraw their data. Grasping and locomotion tasks were counterbalanced across participants.

### Data Analysis

In the grasping task, we were primarily interested in participants’ overall movement times, the lateral hand positions when passing the obstacle, and their overall responsiveness to obstacle position. Movement onset was defined as the moment in time at which one of the finger markers (index finger or thumb) exceeded a velocity threshold of 0.05 mm/ms. Movement offset was determined by using a combination of a spatial criterion and a velocity threshold. Specifically, we first determined the largest distance in *y* direction of the index finger marker during the trial and then searched for the frame with the lowest velocity of both markers ±24 frames (100 ms) from when the hand had reached this position. MD was defined as the time between movement onset and movement offset. MDs that were longer or shorter than each participant’s mean value ±2.5 standard deviations were considered outliers and removed from the analysis. Hand position during grasping was calculated as the virtual midpoint of the position of the thumb and index finger markers in three-dimensional space. From this data, we determined participants’ lateral hand position (*x* direction) at the moment the obstacle was passed (i.e., the moment the virtual midpoint reached the *y* position at which the obstacle was placed). Based on this, we further calculated the overall responsiveness to obstacles by determining the average difference between the lateral hand position when the obstacle was placed either left or right of the midline.

For the locomotion task, we determined how long it took participants to complete the task and how much time they spent looking at the four different obstacles. In this task, MD was defined as the time between leaving the lab until entering back in, and the responsiveness to obstacles was defined as the cumulative obstacle fixation duration. Obstacle fixations were obtained using a frame by frame analysis of the video clips showing both the video recording overlaid with participants’ gaze positions (using the BeGaze™ software) and are reported as the percentage of the overall MD.
